# Study on the influence of campus sports peer model on migrant children’s psychological adaptation, health promotion and urban social integration

**DOI:** 10.3389/fpubh.2026.1870232

**Published:** 2026-06-23

**Authors:** Wenbo Ma, Xiangdong Ding, Nan Zhang

**Affiliations:** 1 Xi’an University of Architecture and Technology, Shaanxi, Xi’an, China; 2 Northwest Minzu University, Gansu, Lanzhou, China; 3 Xi’an University of Architecture and Technology, Shaanxi, Xi’an, China

**Keywords:** campus sports, health promotion, migrant children, peer model, psychological adaptation, urban social integration

## Abstract

**Background:**

This study explores the intervention effects of campus sports peer model on migrant children’s psychological adaptation, healthy development and urban social integration, and analyzes the differences of its effects, so as to provide a basis for the construction of campus assistance system.

**Methods:**

In this study, 420 migrant children in urban public primary and secondary schools were randomly selected and divided into experimental group (*n* = 210) and control group (*n* = 210). At the same time, hierarchical design was carried out according to grade (primary school/junior high school) and floating duration (≤2 years />2 years) to explore the intervention effect of campus sports peer model in different groups. The experimental group implemented peer intervention in campus sports for 12 weeks (including peer-to-peer sports training, team sports competition, etc.), while the control group adopted routine physical education. Through the psychological adaptation scale, physical fitness test and social integration questionnaire system, the intervention effect was compared and analyzed.

**Results:**

Compared with the control group, the total score of psychological adaptation, self-esteem, BMI compliance rate, cardiopulmonary endurance, muscular endurance, regular exercise habit rate, urban belonging and peer acceptance were significantly higher in the experimental group, while the scores of anxiety, depression and perceived discrimination were significantly lower. The stratified study showed that the scores of self-esteem in psychological adaptation, muscular endurance in health promotion and peer acceptance in social integration of migrant children in primary school group were significantly higher than those in junior high school group (*p* < 0.05). The scores of anxiety and city belonging in the group with the duration of migration ≤2 years were better than those in the group with the duration of migration > 2 years (*p* < 0.05).

**Conclusion:**

Campus sports peer model can effectively improve migrant children’s psychological state, improve their health literacy, solve the identity dilemma and build social bridges, and the intervention effect is more prominent for the young and short-term migrant children. It is an effective intervention model to promote migrant children’s psychological adaptation, healthy development and urban social integration, and can provide reference for migrant children’s educational assistance and social support system construction.

## 1 Introduction

With the deepening of China’s new urbanization strategy, the floating population has changed from “individual workers” to “family migration,” and the scale of migrant children has continued to expand. At present, the number of migrant children in China has reached 71.09 million, which has become a special group that cannot be ignored in the process of urbanization (1). From a global perspective, international migrant children are also faced with the difficulties of migration adaptation, mental health and social integration. The psychological stress, health inequality and social exclusion of migrant children have become a common global public health challenge. The issue of migrant children in China is an important part of global migrant health research (2). The adaptation problems encountered in the process of migration are highly common with those of international migrant children, and they are also concentrated in the triple intertwined dilemma of psychological adaptation, healthy development and urban social integration. This group left their original living environment with their parents and entered the unfamiliar urban campus and social scene, facing the triple dilemma of psychological adaptation, healthy development and social integration. The quality of their survival and development is not only related to the lifelong growth of individuals, but also related to educational equity, social harmony and the improvement of the future population quality of the country, which has become a major livelihood issue of common concern in academic circles and policy levels (3). Psychological maladjustment, low health literacy and difficulties in urban social integration do not exist independently, but are mutually causal and mutually reinforcing: negative psychological emotions will reduce the willingness to participate in sports and social initiative; insufficient health level further weakens self-confidence and campus integration ability; the lack of social acceptance in turn intensifies psychological alienation and negative lifestyle, forming a vicious circle. Psychological maladjustment is the primary dilemma faced by migrant children, and it is also the core bottleneck that restricts their healthy growth (4). Migrant children are prone to negative emotions such as anxiety and depression due to sudden changes in their living environment, insufficient parent–child companionship, and vague identity, and their self-esteem level is low, and they are in the psychological state of “marginal people” for a long time (5). The research shows that the scores of depression and anxiety of migrant children are significantly higher than those of urban registered children, and the parent–child relationship and peer relationship are positively correlated with the level of psychological adaptation, and the bad psychological state further aggravates their social adaptation difficulties (6, 7). Children who have stayed behind and gone on the move have more obvious psychological impact brought by drastic environmental changes, and are prone to emotional alienation and social withdrawal. Without effective intervention, their sound personality development will be affected (8). The Action Plan for Strengthening the Care and Protection of Migrant Children clearly puts forward that it is necessary to strengthen the mental health care service for migrant children and solve their psychological adaptation dilemma (9), which provides a clear policy orientation for relevant intervention research.

Low health literacy is a prominent shortcoming in the development of migrant children, which is obviously different from that of urban registered children (10, 11). Migrant children’s families are mostly engaged in informal employment, and their income fluctuates greatly, and their health investment is insufficient. In addition, their parents’ health awareness is weak, which leads to the lag of their healthy lifestyle development, and their BMI compliance rate, cardiopulmonary endurance and other health fitness indicators are significantly lower than those of urban children (12). Therefore, it is necessary to popularize children’s healthy lifestyle, enhance their physical fitness, promote sunshine sports and ensure their healthy development (13, 14). However, at present, the health promotion intervention for migrant children is mainly based on the popularization of single health knowledge, lacking a carrier that is both interesting and effective, and it is difficult to help them develop long-term exercise habits, and the health promotion effect is limited. It is urgent to explore a health intervention model that fits the characteristics of migrant children.

The difficulty of urban social integration is a structural dilemma faced by migrant children. The barriers of household registration and social exclusion make it difficult for them to truly integrate into urban life. Migrant children are easy to encounter implicit discrimination on campus, with low peer acceptance and weak urban belonging, making it difficult to establish a stable social network, resulting in an embarrassing situation of “easy physical integration and difficult psychological integration” (15). Campus is the main scene for migrant children to contact with urban society, and its educational and social functions are very important to solve the dilemma of integration. The campus sports peer model is based on the core principles of peer assistance, pair training and teamwork, and builds an equal interactive platform relying on sports activities, which lowers the social threshold of migrant children and enhances their sense of belonging through peer support (16). Compared with the traditional teacher-led physical education, the peer model is more suitable for the vulnerable situation of migrant children: the teacher-led model focuses on skill transfer and lacks targeted social support and emotional connection; the peer model is based on equal interaction, which meets the three core psychological needs of autonomy, competence and belonging in the theory of self-determination. It can reduce social anxiety and strengthen social support network through peer help, which is more likely to stimulate migrant children’s participation motivation and self-efficacy. At the same time, relying on the social capital theory, it can accumulate campus social capital through peer interaction, make up for the obstacles of integration caused by the lack of family socio-economic status, and realize the coordinated improvement of psychology, health and integration in the low-pressure and high-participation scene. Theoretically, the peer model of campus sports may relieve the psychological pressure of migrant children, improve their physical fitness and weaken social barriers through equal peer interaction, which has potential intervention value, but its actual effect remains to be empirically tested. This model has obvious advantages, which can promote psychological adaptation, physical fitness improvement and social integration with low pressure and high participation, alleviate loneliness, inferiority and other problems, and help develop rule awareness and cooperation ability. At present, it is widely used in physical education class, recess and community activities in primary and secondary schools (17), which has become an effective way to promote the healthy growth of migrant children and their integration into the city. It is both educational, practical and scalable. Campus sports, as an important carrier with both fitness and social attributes, can break down identity barriers and build social bridges, which has unique advantages in promoting teenagers’ mental health, improving their physical fitness and enhancing their sense of belonging to the group, and provides important ideas for solving the triple dilemma of migrant children (18).

The three dilemmas of migrant children’s psychological maladjustment, low health literacy and difficulty in integrating into urban society are intertwined. The existing intervention measures have problems such as insufficient pertinence and single carrier, which are difficult to meet their diversified development needs. Based on the randomized controlled trial design, this study established two research hypotheses: First, compared with the conventional physical education teaching, the campus sports peer model can significantly improve the psychological adaptation, health promotion and the overall level of urban social integration of migrant children; second, the intervention effect has the characteristics of hierarchical heterogeneity in grade and flow duration. The purpose of this study is to empirically test the intervention effect of campus sports peer model, clarify the internal correlation mechanism among psychological adaptation, health promotion and urban social integration, enrich the theoretical system of campus sports intervention for floating children, and provide theoretical basis and empirical support for the research of social support intervention for similar youth groups.

## 2 Methods

### 2.1 Research objects

In this study, multi-stage stratified random sampling method was used, and urban public primary schools (grades 3–6) and urban public junior high schools (grades 7–9) were selected as research sites to screen migrant children who met the inclusion and exclusion criteria as research objects. Migrant children are defined as compulsory education students whose household registration is not in the current city, who have lived in the city with their parents or legal guardians for 6 months or more and have attended the current school for 1 semester.

#### 2.1.1 Inclusion criteria

① 8–15 years old, meeting the school-age requirements of compulsory education. ② The household registration is non-local, and the reason for the mobility is the migration of family workers. ③ No serious organic diseases such as heart, lungs and bones, no contraindications to exercise, and normal participation in sports activities. ④ The guardian informed consent, voluntarily participated in this study and signed the informed consent form. ⑤ During the study period, there was no transfer or suspension from school, and 12 weeks of intervention and various tests were completed.

#### 2.1.2 Exclusion criteria

① Those with a history of mental illness or mental disorder or who are undergoing psychological intervention. ② Long-term use of drugs that affect sports ability and emotional state. ③ During the study period, due to injury, leave of absence and other reasons, the cumulative number of interventions or tests was ≥3 times. ④ Those who have had systematic sports training experience in the past (specialized sports special classes and competitive sports training).

#### 2.1.3 Sample size estimation

Using G*Power 3.1 software to estimate the sample size, taking the psychological adaptation scale as the main outcome indicator, referring to the previous similar research results, setting *α* = 0.05 (bilateral), *β* = 0.10, and the effect amount *d* = 0.40, and considering the drop-off rate of 10%, the sample size is finally determined to be 420. The subjects were divided into experimental group (*n* = 210) and control group (*n* = 210) by random number table method, and at the same time, according to grade (primary school group: 8–12 years old, *n* = 209; junior high school group: 13–15 years old, *n* = 211), the duration of mobility (≤2 years group, *n* = 187; group > 2 years, *n* = 233). The main reasons for sample dropping/eliminating are transfer, lost visit and incomplete evaluation during the intervention period. A total of 37 students were dropped/eliminated, with a dropping rate of 8.81%, which is in line with the preset range of 10%. There is no statistical difference between the two groups in gender, age, grade, duration of mobility, baseline psychological adaptation, healthy physical fitness and social integration level (*p* > 0.05), which is comparable.

The whole process of the study strictly follows the relevant requirements of Helsinki Declaration, and fully protects the privacy, information and voluntary participation rights of the research subjects. All the test data are strictly confidential and are only used for the analysis of this study, and no personal information is disclosed. Ethics Committee endorsed the implementation of this research plan.

### 2.2 Research design

This study adopts randomized controlled trial (RCT) design, the whole process adopts quantitative research design, eliminates qualitative interview links and maintains the single focus of research methodology. The trial period is 16 weeks, including 2 weeks of pre-trial, 12 weeks of formal intervention and 2 weeks of follow-up. The experimental group implemented the peer model of campus sports intervention, while the control group adopted the conventional sports teaching mode. The intervention duration of both groups was 45 min three times a week, and the intervention period was arranged in the afternoon after-school service hours (16:30–18:00) to avoid conflicts with normal teaching and family activities. During the intervention period, the two groups did not increase the amount of physical activity, did not accept other psychological intervention or health promotion related guidance, and ensured the independence of the intervention effect.

#### 2.2.1 Random grouping method

Using simple random sampling method, 420 subjects who meet the standard were divided into experimental group and control group according to the ratio of 1:1. A table of random numbers (range 1–420) was made by a special person, with each number corresponding to one subject. Those with odd numbers were included in the experimental group and those with even numbers were included in the control group. The grouping process adopts blind method, and the grouping personnel do not participate in the follow-up intervention implementation and effect evaluation, and the evaluators do not know the grouping situation of the research objects, so as to ensure the randomness and objectivity of the grouping.

#### 2.2.2 Quality control

Set up a quality control team, composed of three experts in the fields of physical education, psychology and public health, responsible for supervising the implementation of intervention and data collection throughout the process. Before the intervention, all intervention personnel (physical education teachers) should be trained in a unified way, and the intervention process, operating standards and matters needing attention should be clarified. Only after passing the examination can they participate in the intervention; in the grouping and evaluation stage, the strict blind method is implemented: the grouping personnel independently complete the random grouping and do not participate in the follow-up intervention and evaluation; evaluators and statisticians are blind to the grouping situation in the whole process, and only intervening in the implementation of targeted peer-to-peer teaching cannot achieve blindness, which has been clearly explained in the research limitations.

##### 2.2.2.1 Control of confounding factors

During the intervention period, the duration, frequency and venue conditions of the two groups were unified, and the daily physical activity level of the two groups was objectively monitored by accelerometers to ensure that there was no statistical difference in the amount of extra physical activity between the two groups (*p* > 0.05), and the confounding interference caused by the difference in exercise dose was excluded; at the same time, the baseline confounding factors such as family background, previous sports experience and mental health history were controlled to ensure that the other conditions of the two groups were highly consistent except the intervention mode.

##### 2.2.2.2 Process quality control

During the intervention, check the intervention records twice a week to check the intervention duration, content, number of participants and intensity, and correct the irregular operation in time; conduct intervention fidelity verification once every 4 weeks to ensure that the consistency of intervention content and scheme is ≥95%; establish the withdrawal registration system of the research object, record the reasons in detail, and ensure that the withdrawal rate is controlled within the preset range; after the data collection is completed, two special personnel will enter and check the data, respectively, to ensure the accuracy of the data, and verify and supplement the missing and abnormal data. Those who cannot be supplemented will be treated as missing values (multiple imputation).

### 2.3 Intervention measures

#### 2.3.1 Experimental group: peer model intervention in campus sports

The intervention period is 12 weeks, and it is divided into three stages (each stage is 4 weeks). The closed-loop mode of “peer pairing-mutual training-team competition-feedback promotion” is adopted, and the targeted sports activities are designed according to the age characteristics, sports ability and psychological needs of migrant children, as follows:

(1) Peer pairing principle adopts “heterogeneous pairing” mode, and makes reasonable pairing according to the sports ability (basic, medium and good), personality characteristics (introversion and extroversion) and flow duration of the research object, so as to ensure that each pair of peers are complementary in ability and personality. The age difference between each pair of peers in the primary school group is ≤1 year, and the age difference between each pair of peers in the junior high school group is ≤1 year. After pairing, it will be fixed, and a 15-min peer communication time will be arranged once a week to promote mutual understanding and trust. The pairing process is jointly participated by physical education teachers and class teachers, and adjusted according to the wishes of the subjects to ensure the rationality and feasibility of pairing.
(2) Contents of phased intervention: ① The first stage (week 1–4): the adaptation and basic training stage, focusing on basic sports skills training, including queue practice, jogging, skipping rope, standing long jump, etc., focusing on cultivating the sense of cooperation among peers, and arranging 10 min of peer-to-peer mutual help exercises (one-on-one guidance on skipping rope and correcting running posture) in each class, with teachers providing full guidance to help peers establish trust relationships; ② The second stage (week 5–8): in the promotion and cooperative training stage, team sports events, including relay race, tug-of-war, group skipping, fun basketball/football (simplified rules), etc., are added, and 15 min of team cooperation exercises are arranged in each class to guide the peers to cooperate and encourage each other, and cultivate the sense of rules and team cohesion; ③ The third stage (week 9–12): the consolidation and competition stage, in which peer team competitions are organized within classes and between grades. The competition items combine the training contents of the first two stages, set up group awards and individual mutual aid awards, strengthen the support and recognition among peers, and guide the research subjects to sum up the gains in the intervention process and improve their sense of self-efficacy.
(3) Intervention parameters are three times a week, 45 min each time, including 5 min of warm-up (jogging and joint activities), 35 min of main intervention (pair training, team activities, competitions, etc.) and 5 min of relaxation (stretching and deep breathing); the intervention intensity should be controlled at moderate intensity, and it is appropriate that the heart rate of the research object reaches 60–70% of the maximum heart rate (maximum heart rate = 220- age) during exercise. Physical education teachers can monitor the intervention intensity in real time through the heart rate monitor to ensure that the intervention intensity reaches the standard; in the process of intervention, teachers pay attention to the emotional state and physical reaction of the subjects in time, and adjust the training intensity or suspend training for the subjects with fatigue and anxiety in time.

#### 2.3.2 Control group: conventional physical education teaching mode

During the intervention, in addition to subjective reports, both the experimental group and the control group used accelerometers to objectively monitor the daily physical activity level, to ensure that there was no difference in the amount of extra physical activity between the two groups, and to exclude the interference of confounding variables. The control group adopts the school’s regular physical education teaching scheme, which is consistent with the intervention duration and frequency of the experimental group (three times a week, 45 min each time). The teaching content is strictly implemented in accordance with the Compulsory Education Physical Education and Health Curriculum Standard, including basic physical skills training, physical knowledge explanation, regular physical exercise, etc. There is no special peer support link, team competition and psychological guidance. The teaching process is implemented by the school physical education teacher according to the regular teaching process, and no additional intervention measures related to this study are added.

### 2.4 Detection indicators and detection methods

During the intervention, the subjects’ daily moderate and high-intensity physical activity, total activity steps and sitting time were objectively measured by accelerometer GT3x. The wearing requirement was that they should be worn all the time during waking hours (taken off during bathing and sleeping) for 7 days, and the valid data required that they should be worn for ≥10 h and the valid days should be ≥4 days, which was used to objectively quantify the level of physical activity and exclude the confounding factors of exercise dose. Before the intervention (baseline), after the intervention (12th week) and after the follow-up (16th week), the indicators of the two groups were tested, and the testers were trained uniformly and operated in strict accordance with the standardized procedures to ensure the accuracy and comparability of the test results.

#### 2.4.1 Psychological adaptation indicators

Using the Psychological Adaptation Scale for Migrant Children, the Cronbach’s *α* coefficient is 0.89–0.92 and the test–retest reliability is 0.85–0.88, which is suitable for migrant children aged 8–15 (19). There are 42 items in the scale, which are divided into five dimensions: self-esteem (8 items), anxiety (10 items), depression (10 items), loneliness (8 items) and psychological resilience (6 items). Likert 5-point scoring method (1 = completely inconsistent, 5 = completely consistent) is adopted, in which the dimensions of anxiety, depression and loneliness are reverse scores. In this study, the total score of the original scale (42 ~ 210 points) was standardized into 0 ~ 60 zones according to the linear formula, which was convenient for the comparison between groups and the interpretation of the results. The higher the score after standardization, the better the level of psychological adaptation.

#### 2.4.2 Health promotion indicators

Health promotion indicators include healthy physical fitness indicators and exercise habit indicators (20), in which healthy physical fitness indicators adopt standardized detection methods and exercise habit indicators adopt questionnaire survey methods.

① Body mass index (BMI): measured by a height and weight meter (accuracy 0.1 cm, 0.1 kg). Before measurement, the subjects were required to fasten, take off their shoes and wear light clothes. The height was measured in standing posture (feet together, head upright, eyes looking flat in front), and the weight was measured to keep standing stable. BMI = weight (kg) /height^2^ (m^2^). Referring to the BMI classification standard of Chinese school-age children and adolescents, the BMI reached the standard was judged (normal range: 18.5 ≤ BMI < 23.9). ② Cardiopulmonary endurance: A 20 m shuttle run test was adopted, which was implemented according to the standardized process of National Student Physical Health Standards. The test results were converted into standardized endurance scores. The higher the score, the better the cardiopulmonary endurance. ③ In this study, the evaluation index is defined as muscle endurance. In order to avoid methodological bias caused by different gender and age test tools, primary and junior high school girls use 1-min sit-ups and junior high school boys use 1-min push-ups. All the original test times are converted into standard percentile scores according to the National Standards for Students’ Physical Health, and then compared between groups and layers. The selected test items have been verified by the reliability and validity of school-age children and floating children in China, which is suitable for the evaluation and application of this study. (4) Flexibility: The sit-and-reach test was adopted, and the sit-and-reach test tester (with an accuracy of 0.1 cm) was used. The subjects sat with their legs straight and their feet close to the tester, and pushed the cursor forward with both hands, and recorded the maximum pushing distance. The longer the distance, the better the flexibility.

(2) Exercise habit indexes were evaluated using the Adolescent Exercise Habit Self-Rating Scale, which was tested for reliability and validity with Cronbach’s alpha coefficient of 0.86 and test–retest reliability of 0.83. It is suitable for school-age children and adolescents (21). The questionnaire consisted of 18 items, divided into 4 dimensions: exercise frequency (6 items), exercise duration (4 items), exercise type (4 items), and exercise autonomy (4 items). The Likert 4-point score method was used (1 = completely disagreed, 4 = completely agreed). The higher the total score, the better the exercise habit; at the same time, the exercise habit development rate (defined as the proportion of study subjects who exercise ≥3 times a week and exercise ≥30 min each time) was counted.

#### 2.4.3 Urban social integration indicators

Using the Urban Social Integration Scale of Migrant Children for evaluation, revised in combination with the needs of this study, after the reliability and validity test, Cronbach’s alpha coefficient was 0.90, and the test–retest reliability was 0.87. A total of 36 items in the questionnaire were divided into four dimensions: urban belonging (10 items), peer acceptance (8 items), cultural adaptation (8 items), and perceived discrimination (10 items). The Likert 5-point score method was adopted (1 = complete disagreement, 5 = complete agreement), among which the perceived discrimination dimension was reverse score, and the total score of the scale was 25–125 points. The higher the total score, the better the level of urban social integration; the higher the score of each dimension, the better the integration state of the corresponding dimension (the higher the score of the reverse scoring dimension, the weaker the perception of discrimination).

### 2.5 Statistical analysis of data

SPSS 26.0 and AMOS 24.0 were used for data statistical analysis, and SPSS 26.0 was used for normal Shapiro–Wilk test, variance homogeneous Levene’s test, descriptive statistics, independent-samples *t*-test/paired-samples *t*-test, *χ* test, one-way ANOVA and Pearson correlation analysis. The structural equation modeling (SEM) is constructed by using AMOS 24.0 to test the path relationship and internal mechanism among the three latent variables: psychological adaptation, health promotion and urban social integration. The measurement data is expressed by the mean standard deviation (x̄±s), and the counting data is expressed by the rate (%), and the difference is statistically significant with *p* < 0.05. Two-sided test is used in all statistical analysis, and the missing data are processed by multiple interpolation to ensure the reliability of statistical results.

## 3 Results

### 3.1 Baseline data analysis

A total of 420 migrant children were randomly divided into experimental group (*n* = 210) and control group (*n* = 210) according to 1:1, and stratified according to grade (primary school/junior high school) and migrant duration (≤2 years/>2 years). Baseline comparison shows that there are no significant differences between the two groups in gender, age, grade, duration of migration, guardian’s education level, monthly family income and psychological adaptation, physical fitness and urban social integration (all *p* > 0.05), and there is good comparability between the two groups (Table 1).

**Table 1:** Comparison of baseline demographic characteristics and main outcome indicators between two groups of migrant children (M ± SD, n/%).

Index	Experimental group (*n* = 210)	Control group (*n* = 210)	*χ*^2^/t	*p*-value
Gender (M/F)	112/98(53.33/46.67)	108/102(51.43/48.57)	0.152	0.697
Age (years)	10.40 ± 1.82	10.46 ± 1.79	0.326	0.745
Grade (primary/junior)	106/104(50.48/49.52)	103/107(49.05/50.95)	0.091	0.763
Duration of mobility (≤2 years/>2 years)	95/115(45.24/54.76)	92/118(43.81/56.19)	0.09	0.764
Guardian’s education level (junior high school and below/high school and above)	136/74(64.76/35.24)	141/69(67.14/32.86)	0.271	0.603
Standardized total psychological adaptation score	30.52 ± 5.36	24.18 ± 6.02	1.215	0.225
Anxiety score	11.38 ± 2.91	11.51 ± 2.85	0.462	0.644
Depression score	11.48 ± 3.06	11.60 ± 3.11	0.401	0.688
Self-esteem score	9.12 ± 2.18	9.07 ± 2.21	0.236	0.814
Cardiopulmonary endurance	40.12 ± 5.93	39.98 ± 5.86	0.251	0.802
Muscular endurance	32.15 ± 5.02	32.08 ± 4.97	0.147	0.883
A sense of city belonging	20.05 ± 4.31	19.96 ± 4.28	0.214	0.831
Peer acceptance	13.22 ± 2.82	13.18 ± 2.79	0.15	0.881
Perceived discrimination	10.25 ± 2.20	10.33 ± 2.17	0.382	0.703

The baseline comparison between the two groups was *p* > 0.05, which was comparable. The total score of psychological adaptation was standardized (original score 42–210 points, standardized 0–60 points).

Shapiro–Wilk normality test and Levene variance homogeneity test are all above 0.05, which meet the application premise of parameter test. Independent sample T, variance analysis and other parameter statistical methods can be used (see Table 2).

**Table 2:** Test results of normality and variance homogeneity of each observation index.

Observation indicators	Shapiro–Wilk normality test (*p*-value)	Levene homogeneity of variance test (*p*-value)
Total psychological adaptation score	0.216	0.352
Self-esteem score	0.189	0.415
Anxiety score	0.253	0.386
Depression score	0.207	0.431
Cardiopulmonary endurance	0.175	0.368
Muscular endurance	0.232	0.394
BMI up to standard	0.261	0.407
Exercise habit development rate	0.193	0.425
A sense of city belonging	0.228	0.379
Peer acceptance	0.245	0.363
Perceived discrimination	0.201	0.418

### 3.2 Comparison of psychological adaptation indexes between the two groups after intervention

**Figure 1:**
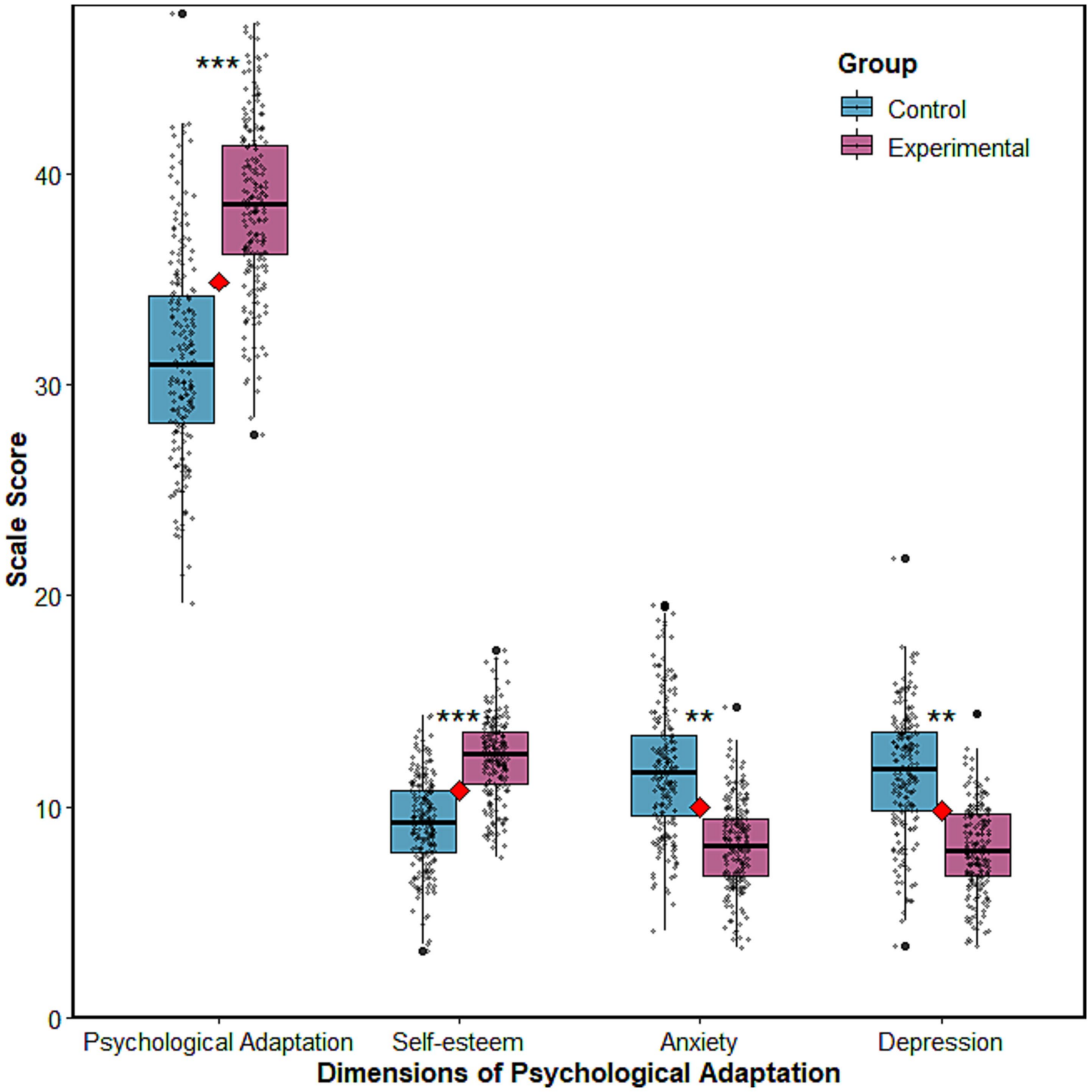
Comparison of scores of psychological adaptation dimensions between two groups of migrant children after intervention. Compared with the control group, **p < 0.01, ***p < 0.001.

After 12 weeks of intervention, the overall level of psychological adaptation and positive emotions in the experimental group were significantly better than those in the control group, and the negative emotions were significantly reduced (Figure 1). The total score of psychological adaptation in the experimental group (38.62 ± 4.15) was significantly higher than that in the control group (31.28 ± 5.03, *p* < 0.001). The self-esteem score (12.35 ± 1.89) was significantly higher than that of the control group (9.18 ± 2.13, *p* < 0.001). The scores of anxiety (8.21 ± 2.05) and depression (7.86 ± 1.98) were significantly lower than those of the control group (11.45 ± 2.87 and 11.54 ± 3.02, all *p* < 0.01).

### 3.3 Comparison of health promotion indicators between the two groups after intervention

**Figure 2:**
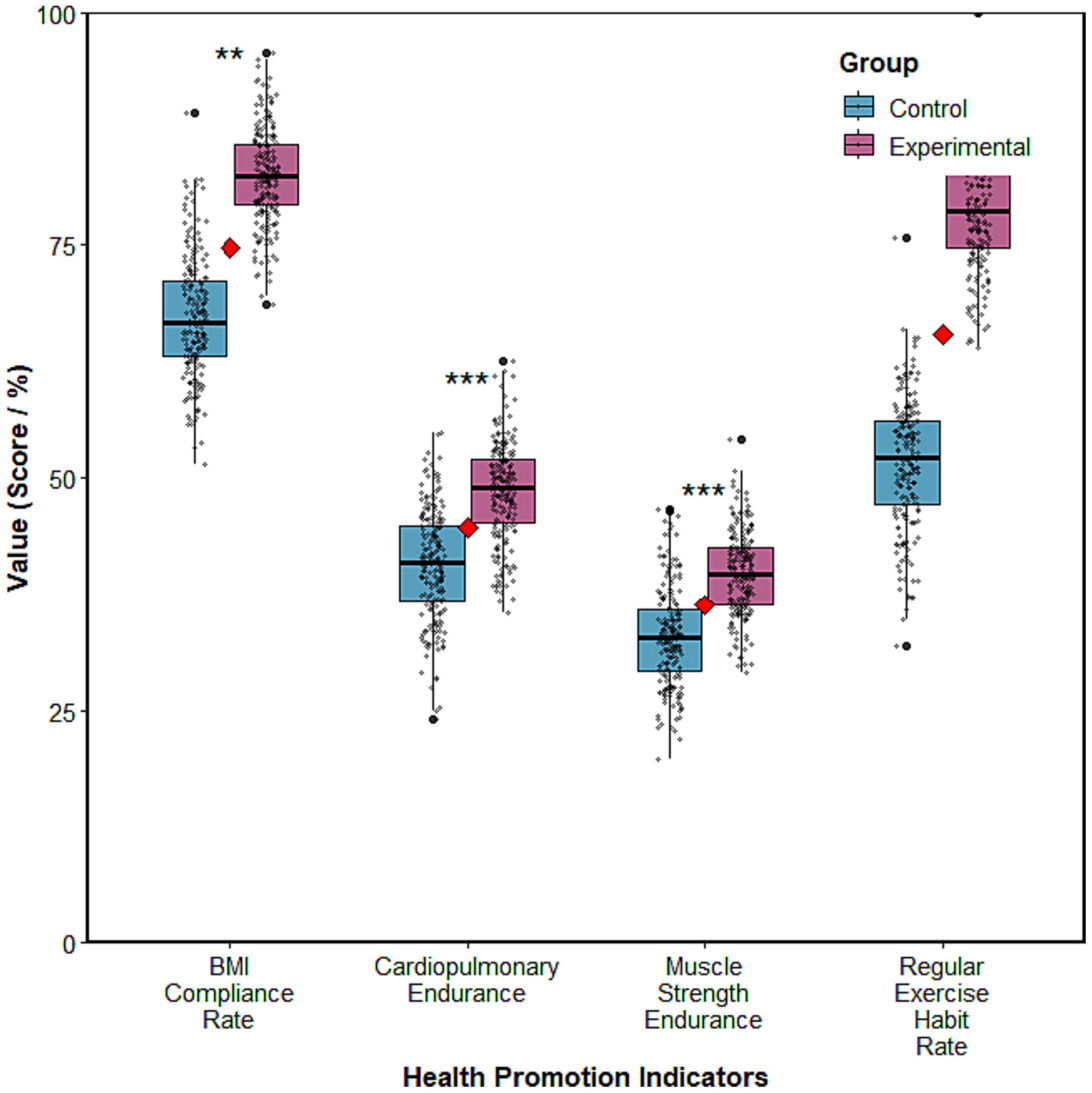
Comparison of health promotion indicators between two groups of migrant children after intervention. Compared with the control group, **p < 0.01, ***p < 0.001. Including indicators: BMI compliance rate, cardiopulmonary endurance, muscular endurance, and regular exercise habit rate.

In terms of healthy physical fitness and healthy behavior, many objective indicators of the experimental group were significantly better than those of the control group (Figure 2). BMI compliance rate: 173/210 (82.38%) in the experimental group and 141/210 (67.14%) in the control group, with significant difference (*p* < 0.01); cardiopulmonary endurance: Using the 20 m round-trip run test and converted to standardized endurance scores, the experimental group (48.56 ± 5.23) was significantly higher than the control group (40.54 ± 5.87, *p* < 0.001); muscle endurance: The experimental group (39.78 ± 4.51) was significantly higher than the control group (32.46 ± 4.98, *p* < 0.001); the rate of regular exercise habits was 165/210 (78.57%) in the experimental group and 108/210 (51.43%) in the control group, with significant difference (*p* < 0.01).

### 3.4 Comparison of indicators of urban social integration between the two groups after intervention

**Figure 3:**
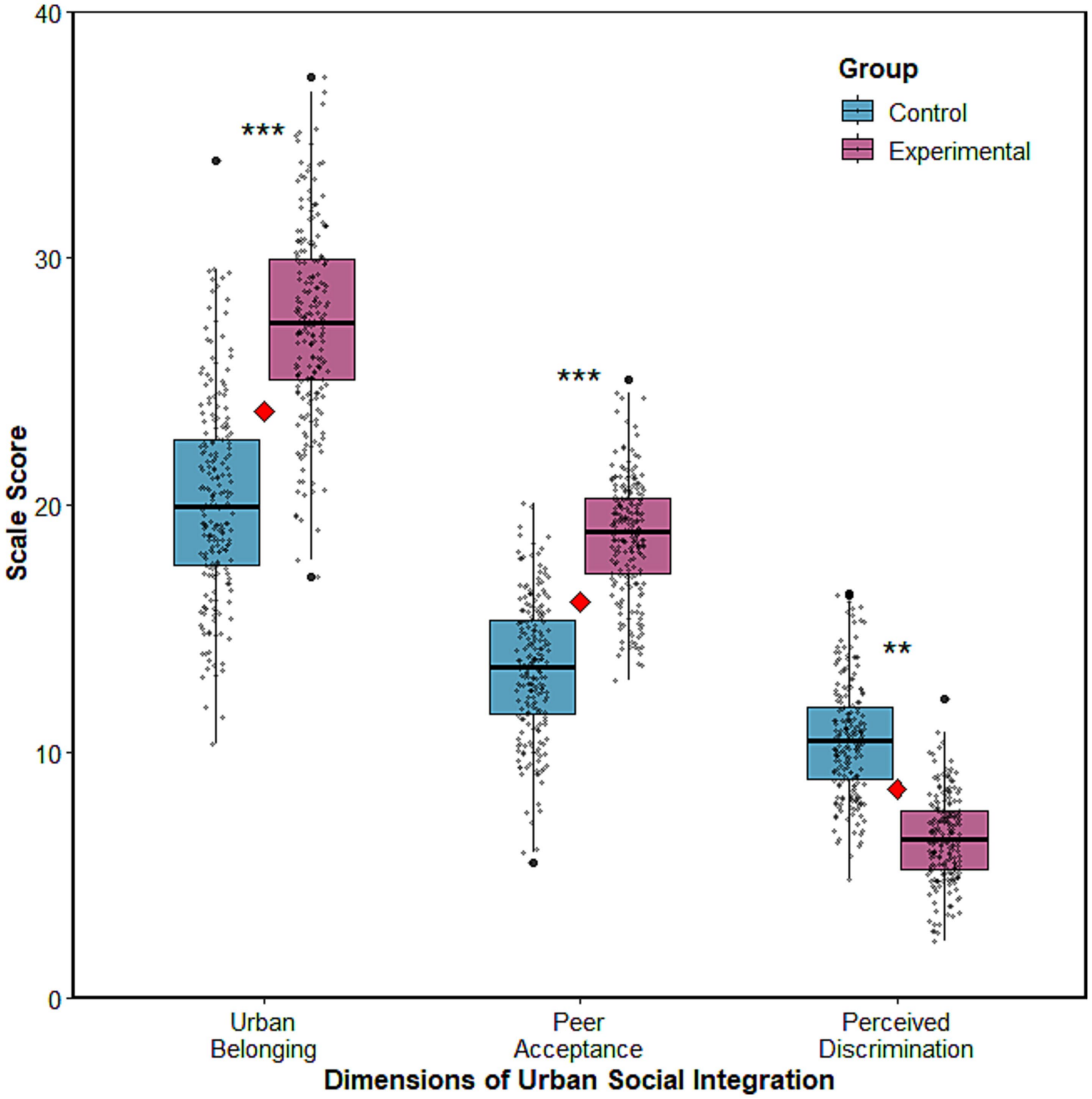
Comparison of scores of various dimensions of urban social integration between two groups of migrant children after intervention. Compared with the control group, **p < 0.01, ***p < 0.001.

In terms of social integration, the urban belonging and peer acceptance in the experimental group were significantly improved, and the perception of discrimination was significantly reduced. The urban belonging in the experimental group (27.35 ± 3.89) was significantly higher than that in the control group (20.17 ± 4.26, *p* < 0.001). Peer acceptance (18.76 ± 2.34) was significantly higher than that of the control group (13.29 ± 2.78, *p* < 0.001). Perception of discrimination (6.52 ± 1.76) was significantly lower than that of the control group (10.30 ± 2.15, *p* < 0.01; see Figure 3).

### 3.5 Hierarchical analysis of grade and flow duration

#### 3.5.1 Grade stratification

Table 3 shows that the scores of self-esteem, muscular endurance and peer acceptance of migrant children in different grades in primary school group are higher than those in junior high school group, and the data are presented as mean standard deviation, in which the self-esteem in primary school group is 13.12 ± 1.78, muscular endurance is 41.23 ± 4.35, and peer acceptance is 19.87 ± 2.16, and the corresponding indexes in junior high school group are 11.58 ± 1.92, respectively. *t*-test results of independent samples showed that there were significant differences in self-esteem (*t* = 2.126, *p* = 0.034), muscular endurance (*t* = 2.315, *p* = 0.021) and peer acceptance (*t* = 2.208, *p* = 0.028) between the two groups (all *p* < 0.05).

**Table 3:** Comparison of main intervention indexes of migrant children in different grades (M ± SD).

Index	Primary school group (*n* = 209)	Junior high school group (*n* = 211)	*t*-value	*p*-value
Having self-respect	13.12 ± 1.78	11.58 ± 1.92	2.126	0.034
Muscle endurance	41.23 ± 4.35	38.31 ± 4.67	2.315	0.021
Peer acceptance	19.87 ± 2.16	17.65 ± 2.41	2.208	0.028

#### 3.5.2 Stratification of flow duration

From Table 4, it can be seen that there are obvious differences between anxiety and urban belonging index of migrant children with different migration duration. The anxiety score (7.89 ± 1.96) of the group with the floating time ≤2 years is lower than that of the group with the floating time >2 years (8.53 ± 2.11), and the city belonging score (29.12 ± 3.67) is higher than that of the group with the floating time >2 years (25.58 ± 3.94). *t*-test of independent samples showed that there were significant differences in anxiety (*t* = 2.074, *p* = 0.039) and sense of urban belonging (*t* = 2.183, *p* = 0.030) between the two groups (all *p* < 0.05), which indicated that migrant children with less than 2 years had lower anxiety level and stronger sense of urban belonging, which was significantly better than those with more than 2 years.

**Table 4:** Comparison of main intervention indexes of migrant children with different migration duration (M ± SD).

Index	Flow ≤2 years (*n* = 187)	Flow >2 years (*n* = 233)	*t*-value	*p*-value
Anxious	7.89 ± 1.96	8.53 ± 2.11	2.074	0.039
Urban sense of belonging	29.12 ± 3.67	25.58 ± 3.94	2.183	0.03

### 3.6 Pearson correlation analysis of psychological adaptation, health promotion and urban social integration

**Figure 4:**
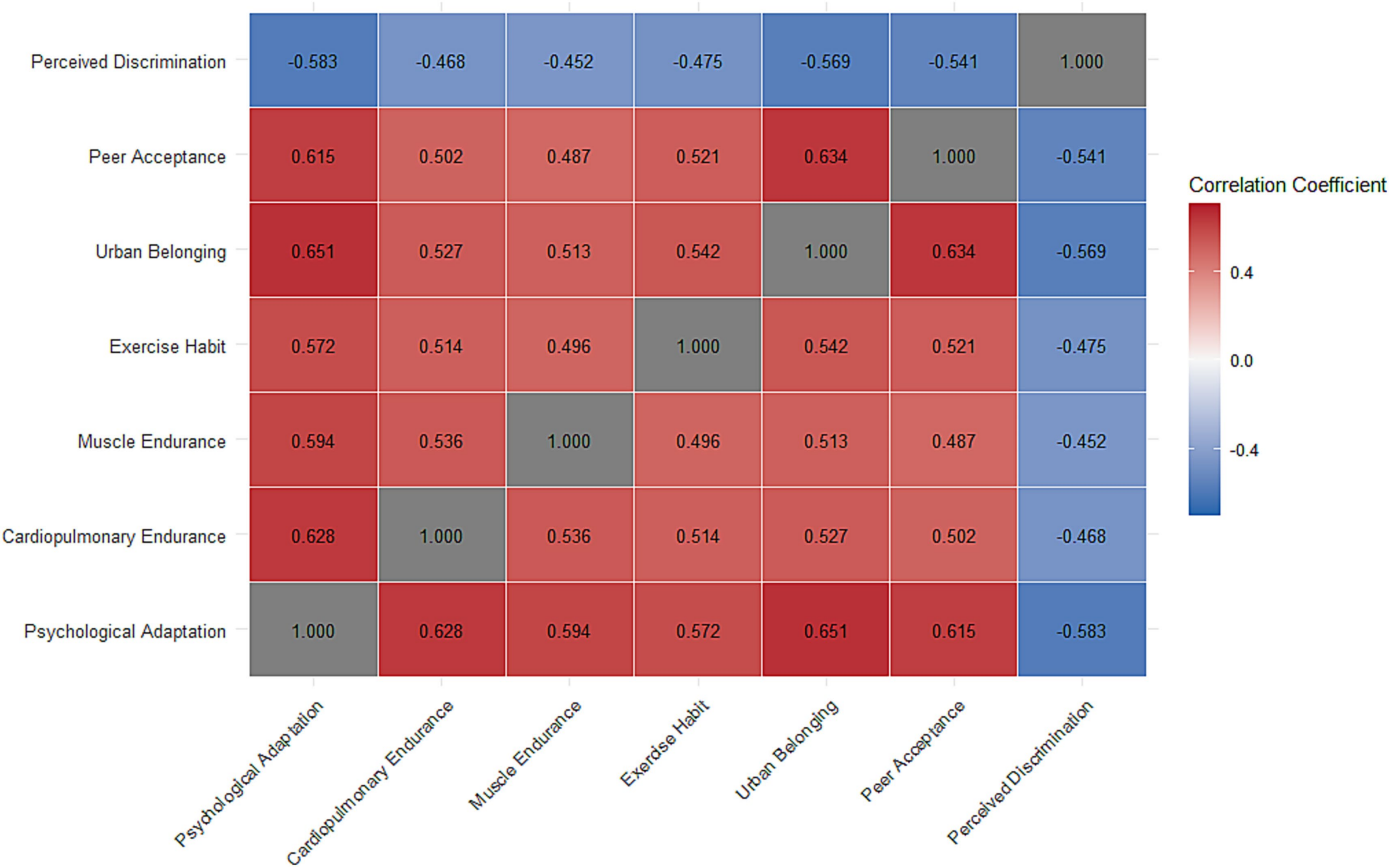
Pearson correlation coefficient heat map of migrant children’s psychological adaptation, health promotion and urban social integration.

The total score of psychological adaptation of migrant children was positively correlated with cardiopulmonary endurance, muscular endurance and the rate of forming exercise habits (*r* = 0.572 ~ 0.628, *p* < 0.01). The total score of psychological adaptation was positively correlated with the urban belonging and peer acceptance (*r* = 0.615 ~ 0.651, *p* < 0.01), and negatively correlated with discrimination perception (*r* = −0.583, *p* < 0.01). The indexes of fitness and exercise habit of health promotion are also moderately significantly correlated with the dimensions of urban social integration (see Table 5; Figure 4).

**Table 5:** Pearson correlation coefficient matrix of psychological, health and social integration dimensions.

Index	Total psychological adaptation score	Cardiopulmonary endurance	Muscular endurance	Exercise habit development rate	A sense of city belonging	Peer acceptance	Perceived discrimination
Total psychological adaptation score	1.000	0.628	0.594	0.572	0.651	0.615	−0.583
Cardiopulmonary endurance	0.628	1.000	0.536	0.514	0.527	0.502	−0.468
Muscular endurance	0.594	0.536	1.000	0.496	0.513	0.487	−0.452
Exercise habit development rate	0.572	0.514	0.496	1.000	0.542	0.521	−0.475
A sense of city belonging	0.651	0.527	0.513	0.542	1.000	0.634	−0.569
Peer acceptance	0.615	0.502	0.487	0.521	0.634	1.000	−0.541
Perceived discrimination	−0.583	−0.468	−0.452	−0.475	−0.569	−0.541	1.000

## 4 Discussion

In the process of urbanization, family migration has become the main form of population mobility, but migrant children still face multiple pressures in environmental adaptation, social support and identity, and are prone to psychological adaptation problems such as anxiety, depression and low self-esteem (22, 23). Previous studies have pointed out that migrant children’s psychological stress level is significantly higher than that of local children due to differences in household registration, cultural barriers, unstable peer relationship and other factors, and long-term negative emotions will directly affect their academic development and personality integrity (24). This study found that, compared with the conventional physical education teaching, the campus sports peer model can significantly improve the total score of psychological adaptation and self-esteem, and reduce the scores of anxiety and depression, suggesting that sports intervention centered on peer help can effectively alleviate psychological difficulties. The internal mechanism may be as follows: on the one hand, moderate-intensity regular exercise can improve emotional state and reduce stress response by regulating the secretion of neurotransmitters such as endorphins and dopamine (25); on the other hand, objective health improvement, such as reaching the standard of BMI, improving cardiopulmonary endurance and muscular endurance, will strengthen the positive experience brought by exercise, further promote the continuous secretion of dopamine and endorphins, improve the regulation of neurotransmitters in the body, promote positive emotions and enhance self-esteem, and the optimization of health status will be transformed into the promotion of self-worth and psychological resilience (26).

The results of this study are in line with the theory of self-determination, and the model of peer assistance gives migrant children the right to choose their own sports partners and participate in team decision-making to meet their independent needs. Cooperate to complete sports tasks, improve sports competence and meet their competence needs; stable peer interaction builds an emotional support network to meet their belonging needs, and the satisfaction of the three psychological needs jointly drives the improvement of psychological adaptation; at the same time, according to the theory of social capital, the campus social capital accumulated by peer interaction provides emotional support, information support and behavior demonstration for migrant children, makes up for the lack of family social capital and becomes an important protective factor for psychological adaptation; peer relationship, as an important source of social support, can make up for the risks caused by the lack of social and economic status of families and provide continuous protection for psychological adaptation (27, 28).

In the dimension of health promotion, this study confirmed that the campus sports peer model can significantly improve the physical fitness level and health behavior of migrant children. The experimental group is significantly better than the control group in BMI compliance rate, cardiopulmonary endurance, muscular endurance and regular exercise habit rate. Migrant children generally have problems such as lack of sports opportunities, low health literacy and weak sports habits. It is difficult to achieve sustained behavior changes simply by relying on regular physical education courses. The peer model significantly enhances the interest and persistence of sports through peer supervision, group competition and cooperative practice, and transforms passive participation into active behavior (29). Regular exercise can not only improve body composition, enhance muscle and cardiopulmonary function, but also promote the formation of healthy lifestyles such as regular work and rest and reasonable diet through positive behavior cycle (30). More importantly, the improvement of health status can further enhance self-efficacy, form a virtuous circle of “sports-health-psychological positivity,” and provide a physiological basis for the all-round development of migrant children.

Urban social integration is the core symbol of migrant children’s adaptation to urban life, while peer acceptance, urban belonging and perceived discrimination are the key indicators to measure social integration. The results of this study show that the peer intervention model can significantly improve the sense of belonging and peer acceptance in the city and reduce the perception of discrimination, indicating that sports activities are an effective carrier to break the group barrier and promote social integration. Sports scenes have natural equality and cooperation, which can weaken the differences in household registration and family background, and enable migrant children to establish trust and friendship under the common goal. Positive peer interaction can reduce loneliness and exclusion, enhance recognition and attachment to the urban environment, and promote the transition from “marginal adaptation” to “active integration.” This result suggests that campus physical education should not only be regarded as a physical exercise course, but also as an important strategy to promote social integration and build an inclusive campus environment.

The results of stratified analysis further reveal the difference characteristics of intervention effects. The improvement of self-esteem, muscular endurance and peer acceptance of migrant children in primary school group is significantly better than that in junior high school group, while children who have been migrant for less than 2 years have better effects in relieving anxiety and improving their urban belonging. The reason is that the primary school is in the critical period of social ability, personality development and exercise habit development, and the psychological plasticity is stronger. Peer support is easy to establish stable and positive peer relationship. However, junior high school students are faced with multiple pressures such as puberty development, academic pressure, more complicated social evaluation, etc. The psychological defense is stronger, and it is more difficult to establish emotional connection in peer interaction. At the same time, academic burden reduces the time of sports participation, resulting in weaker intervention effect than primary school group (31). For children with short mobility, their adaptation mode has not been solidified, and early intervention can effectively block the negative adaptation trajectory and avoid the psychological cumulative effect brought by long-term mobility. The above results have important practical significance, and suggest that campus intervention should adhere to the principles of early detection, early intervention and early support, and give priority to providing accurate peer support services for lower grades and newly immigrated migrant children.

There are some limitations in this study. First, the intervention period is only 12 weeks, the follow-up period is only 2 weeks, and no long-term follow-up of 6 months or more is carried out, so the long-term sustainability and long-term benefit of the intervention effect cannot be verified; second, only accelerometer is used to objectively monitor the level of physical activity, and physiological indexes such as cortisol and heart rate variability are not used to objectively support the psychological adaptation state. The mechanism discussion is still mainly based on subjective scales and behavioral indexes; third, the intervention implementation teachers cannot realize the blind method because of the teaching needs, and there may be slight implementation bias and expectation effect; fourthly, the research object only selects migrant children from public primary and secondary schools in cities, and does not include different schools such as private schools and schools for migrant children. The representativeness of the sample has certain limitations, so the extrapolation of conclusions needs to be cautious. In the future, the follow-up period can be extended to 6–12 months, and the study of the mechanism of action can be deepened by combining physiological indicators and objective biomarkers, the range of sample sources can be expanded, and the age adaptation and school type adaptation scheme of peer patterns can be optimized, so as to further enhance the scientificity and popularization of the research conclusions.

In summary, the campus sports peer model is a comprehensive intervention strategy with low cost, high effect and easy promotion, which can promote the psychological adaptation, healthy development and urban social integration of migrant children simultaneously, improve the campus support system of migrant children, promote educational equity and children’s healthy development.

## 5 Conclusion

In this study, a randomized controlled trial design was used to explore the intervention effect and hierarchical influence of campus sports peer model on migrant children aged 8–15. The results show that this model can significantly improve the psychological adaptation level of migrant children (improving self-esteem and reducing negative emotions), improving health promotion effect (improving physical fitness and cultivating regular exercise habits) and urban social integration ability (improving sense of belonging and peer acceptance and reducing perceived discrimination), and the differences are statistically significant (*p* < 0). Hierarchical analysis shows that the improvement effect of self-esteem, muscular endurance and peer acceptance in the primary school group is better than that in the junior high school group, and the improvement effect of anxiety level and city belonging in the group with floating time ≤2 years is better than that in the group with floating time >2 years (all *p* < 0.05). To sum up, this model can be used as an effective way to promote the physical and mental health and social integration of migrant children, and intervention strategies should be optimized according to the differences of grades and floating time.

## Data Availability

The original contributions presented in the study are included in the article/supplementary material, further inquiries can be directed to the corresponding author.
